# The RAAS Axis and SARS-CoV-2: From Oral to Systemic Manifestations

**DOI:** 10.3390/medicina58121717

**Published:** 2022-11-24

**Authors:** Minela Aida Maranduca, Calin George Vamesu, Daniela Maria Tanase, Andreea Clim, Ilie Cristian Drochioi, Alin Constantin Pinzariu, Nina Filip, Nicoleta Dima, Ionut Tudorancea, Dragomir Nicolae Serban, Ionela Lacramioara Serban

**Affiliations:** 1 Department of Morpho-Functional Sciences II, Discipline of Physiology, “Grigore T. Popa” University of Medicine and Pharmacy, 700115 Iasi, Romania; 2 Internal Medicine Clinic, “St. Spiridon” County Clinical Emergency Hospital, 700115 Iasi, Romania; 3 Department of Internal Medicine, “Grigore T. Popa” University of Medicine and Pharmacy, 700115 Iasi, Romania; 4 Department of Oral and Maxillofacial Surgery and Reconstructive, Faculty of Dental Medicine, “Grigore T. Popa” University of Medicine and Pharmacy, 700020 Iasi, Romania; 5 Department of Morpho-Functional Sciences II, Discipline of Biochemistry, “Grigore T. Popa” University of Medicine and Pharmacy, 700115 Iasi, Romania; 6 Cardiology Clinic, “St. Spiridon” County Clinical Emergency Hospital, 700111 Iasi, Romania

**Keywords:** RAAS, ACE2, SARS-CoV-2, COVID-19

## Abstract

One of the essential regulators of arterial blood pressure, the renin-angiotensin-aldosterone system (RAAS) seems to be one of the most complex mechanisms in the human body. Since the discovery of its key components and their actions, new substances and functions are still being unraveled. The main pathway begins with the secretion of renin in the kidney and culminates with the synthesis of angiotensin II (Ang II)—a strong vasoconstrictor—thanks to the angiotensin-converting enzyme (ACE). Research conducted in 2000 identified another enzyme, named ACE2, that converts Ang II into Ang-(1–7), a heptapeptide with opposing effects to those of Ang II: vasodilation and anti-inflammatory properties. This particular enzyme became of paramount importance during the last two decades, as a result of the confrontation of the human race with life-threatening epidemics. Multiple studies have been performed in order to uncover the link between ACE2 and human coronaviruses, the results of which we systemized in order to create an overview of the pathogenic mechanism. Human coronaviruses, such as SARS-CoV and SARS-CoV-2, attach to ACE2 via their spike proteins (S), causing the destruction of the enzyme. Because ACE2 limits the production of Ang II (by converting it into Ang-(1–7)), its destruction leads to a dysregulated inflammatory response. The purpose of this review is to decipher the complex pathophysiological mechanisms underlying the multiorgan complications (oral, cardiac, pulmonary, systemic) that appear as a result of the interaction of the SARS CoV-2 virus with the angiotensin-converting enzyme type 2.

## 1. Introduction

The emergence of the coronavirus disease 2019 (COVID-19) pandemic determined a radical transformation of both people’s lifestyle and the healthcare systems worldwide. Thus, the international scientific community was put in the situation to react promptly and efficiently, rapidly engaging in unprecedented research efforts, in order to accelerate the clinical studies needed to uncover the pathophysiology of the severe acute respiratory disease coronavirus 2 (SARS-CoV-2) infection.

Even though the majority of confirmed cases are mild, some progress towards a more severe evolution, leading to oral cavity manifestations, inflammatory lung damage, acute respiratory distress syndrome (ARDS), a hypercoagulative state and multiorgan failure. This article follows the interaction between SARS-CoV-2 and its receptor, angiotensin converting enzyme 2 (ACE2), leading to viral entry in the cells that express this molecule on their membrane. The virus can directly injure these cells, and multiple other organs and systems through inflammation. We review the current literature and summarize the proposed pathophysiological mechanisms that turn the host response to SARS-CoV-2 infection into a dysregulated inflammatory state.

Methods: The literature search was conducted in PubMed, Medline and Web of Science. The following words were searched for: RAAS, ACE2, oral mucosa lesion, oral manifestations, COVID-19, and SARS-CoV-2. Duplicate articles were eliminated. Inclusion and exclusion criteria were applied.

## 2. The Renin-Angiotensin-Aldosterone System

Blood pressure is under the control of four parallel pathways, all of which regulate circulating volume through natremia—the concentration of plasma sodium (Na^+^) levels: the renin-angiotensin-aldosterone system (RAAS), the sympathetic nervous system, arginine-vasopressin (antidiuretic hormone—ADH), and atrial natriuretic peptide (ANP) [1].

Renin, the starting point of RAAS, was first discovered in 1898 by Finnish physiologist Robert Tigerstedt and Per Bergman, his student, through experiments conducted at the Karolinska Institute in Sweden. However, as the results could not be repeated by other scientists, it needed to be rediscovered in the 1930s [2]. Renin is an enzyme synthesized in its inactive form, prorenin, by the juxtaglomerular cells of the kidney—modified smooth muscle cells located in the wall of the afferent arteriole. When blood pressure drops, the cells of the macula densa (also a component of the juxtaglomerular apparatus) sense the decrease of the Na^+^ concentration, and in response produce a number of paracrine mediators that activate the release of renin, created due to the splitting of prorenin molecules [3,4,5].

**Figure 1:**
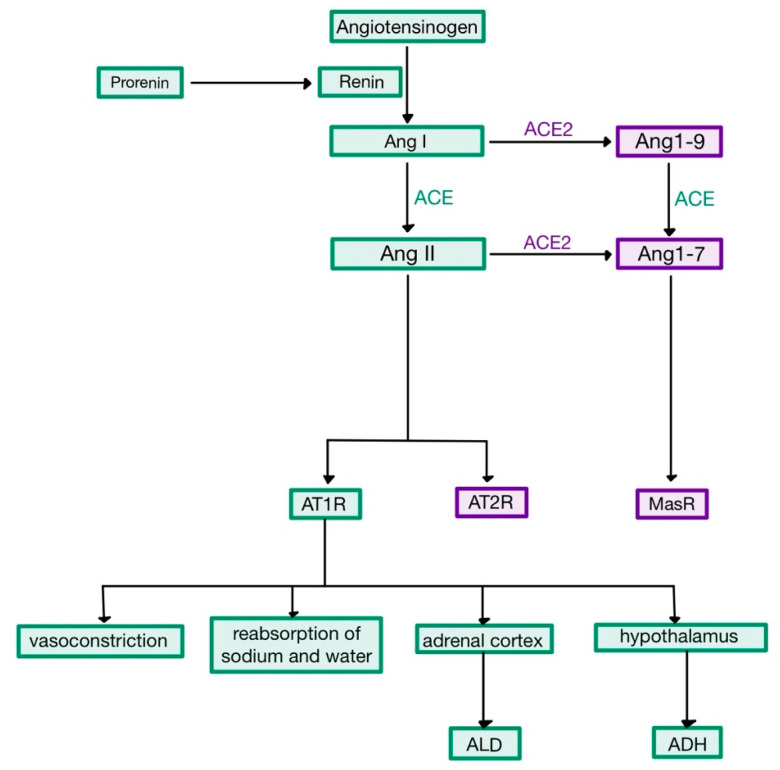
The renin-angiotensin system. ACE, angiotensin converting enzyme; ACE, angiotensin converting enzyme 2; Ang, angiotensin; ALD, aldosterone; ADH, antidiuretic hormone. Green boxes indicate the pro-inflammatory pathway, while purple boxes indicate the anti-inflammatory arm (adapted after [4,6]).

Renin acts enzymatically on a plasma α_2_-globulin named the renin substrate or angiotensinogen (AGT), released by the liver into the systemic circulation [1]. The reaction leads to the creation of angiotensin I (Ang I), a decapeptide with mild vasoconstrictor properties. When Ang I travels through the pulmonary capillaries, it comes in contact with angiotensin converting enzyme (ACE)—present on the endothelial cells. The enzyme cleaves two amino-acids from Ang I, to form the octapeptide angiotensin (Ang) II (Figure 1) [5,6].

**Figure 2:**
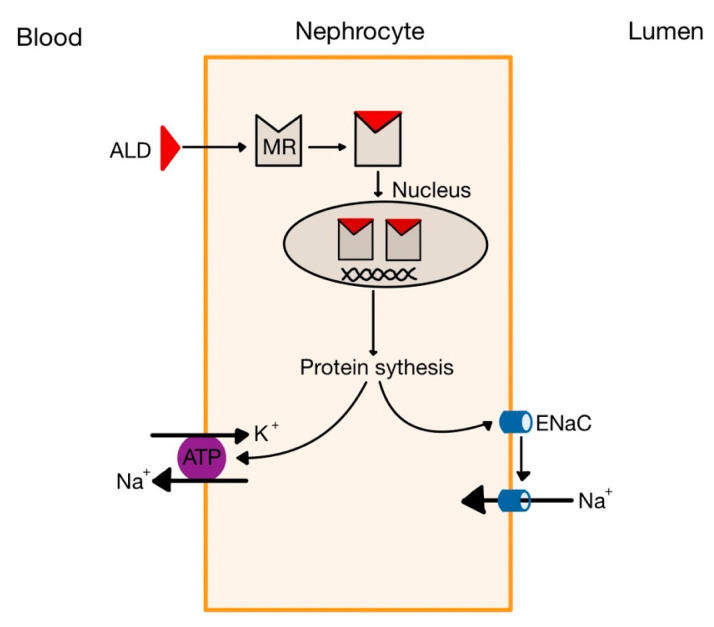
Effects of aldosterone in the kidney. MR, mineralocorticoid receptor; ATP, Na^+^-K^+^ ATP-ase (pump); ENaC, epithelial Na^+^ channels (adapted after [6]).

Angiotensin II is an extremely powerful vasoconstrictor of both renal and systemic vessels, causing an increase of the total peripheral resistance. The mechanism involves the interaction of Ang II with its receptor, AT_1_R, a G protein-coupled receptor which activates phospholipase C, increasing the intracellular concentration of Ca^2+^ and promoting the contraction of vascular smooth muscle cells. At high levels, it causes constriction on the efferent arteriole of the renal glomerulus—much more than on the afferent arteriole—thus reducing the hydrostatic pressure in the tubular capillaries, favouring the reabsorption of Na^+^ and water, which elevates the circulating volume. Moreover, Ang II stimulates the endocrine cells in the zona glomerulosa of the adrenal cortex to produce more aldosterone (ALD). This hormone acts on the principal cells of the collecting ducts by binding to cytoplasmic mineralocorticoid receptors (MR), which stimulate transcription and translation leading to the synthesis of apical Na^+^ and K^+^ channels and basal Na^+^-K^+^ pumps (Figure 2). Therefore, ALD stimulates the reabsorption of Na^+^ and the secretion of K^+^, accompanied by the reabsorption of water (as Na^+^ is an osmotic active substance), also contributing to the elevation of circulating volume [1,7,8].

In addition to the aforementioned effects, Ang II also stimulates the hypothalamic thirst centre, causing an increased production of ADH by the supraoptic and paraventricular nuclei, which is then released by the posterior pituitary gland into the bloodstream. ADH binds to V_2_ receptors in the distal and collecting tubules, increasing both the synthesis and the exocytosis from cytoplasmatic vesicles of aquaporin 2 (AQP-2), thus increasing the cells’ permeability and the reabsorption of water [1,3,9,10].

Besides its role in haemodynamic and hydro-electrolytic balance, Ang II was more recently demonstrated to cause generation of oxidative radicals via AT_1_R and to be involved in inflammatory processes. Ang II also acts on AT_2_R, which mediate vasodilation and nitric oxide (NO) release, having opposite effects to the stimulation of AT_1_R [11,12].

## 3. ACE2

ACE2, a type I transmembrane glycoprotein [13,14], was first discovered in 2000 [2]. The protein is made up of 805 amino-acids, its genes being located on the X chromosome (Xp22). It acts as a carboxypeptidase that cleaves one amino-acid from Ang I to form Ang 1–9, and also from Ang II (its major substrate), creating Ang 1–7 [12,15,16]. Therefore, ACE2 controls the anti-inflammatory arm of RAAS, as it turns Ang II into a heptapeptide with opposing effects, Ang 1–7, hence limiting the interaction of Ang II with AT_1_R and its effects. Ang 1–7 binds to the Mas receptor, a G protein-coupled receptor implicated in leukocyte recruitment, inflammation and vasodilation (Figure 1) [17,18,19,20].

In addition to its physiological functions, ACE2 plays an important role in viral infections, being the receptor for human coronaviruses, including SARS-CoV and SARS-CoV-2. Like the other components of RAAS, ACE2 is ubiquitous [18,21]. ACE2 is expressed on both ciliated and goblet cells of the upper respiratory epithelium (nasopharynx, bronchi, paranasal sinuses), as well as on type 2 pneumocytes and alveolar macrophages in the lungs. Thus, the respiratory system is the main portal of entry for coronaviruses [22,23]. Another important location of ACE2 is the gastrointestinal tract, which is an explanation for the diarrhoea and digestive symptoms seen in patients infected with SARS-CoV-2 [18,24]. High ACE2 expression was also discovered in the surface epithelial cells of the oral mucosa and exfoliated epithelial cells in the saliva, its localisation in the taste buds being a plausible cause for more common COVID-19-related symptoms, such as ageusia (loss of taste) [25,26].

ACE2 is also present in the heart—in pericytes and the endothelium of coronary arteries, and its expression is increased in patients with heart failure. Levels of ACE2 are higher in the lungs of individuals with pulmonary arterial hypertension, as well as present or past smokers. The enzyme is also elevated in patients with cardiovascular diseases, obesity and type 2 diabetes mellitus. These changes in the expression of ACE2 seem to be the reason of the higher mortality rates in COVID-19 patients with comorbidities. ACE2 expression also increases with age, being very low in the lung cells of infants and children, in concordance with the mortality rates of COVID-19 by age [22,26,27,28].

## 4. SARS-CoV-2

Coronaviruses are named for the crown-like appearance of the virions in electron microscopy. After rhinoviruses, they represent the second most prevalent aetiologic agent of the common cold. However, these viruses are known to cause more severe infectious diseases, such as the severe acute respiratory syndrome (SARS), the Middle East respiratory syndrome (MERS), and more recently the coronavirus disease 2019 (COVID-19) [29,30,31].

Human coronaviruses (HCoVs) are classified serologically into four groups: alpha-coronaviruses, beta-coronaviruses, gamma-coronaviruses and delta-coronaviruses. All three of the “pandemic” HCoVs (SARS-CoV, MERS-CoV and SARS-CoV-2) are beta-coronaviruses [32,33].

**Figure 3:**
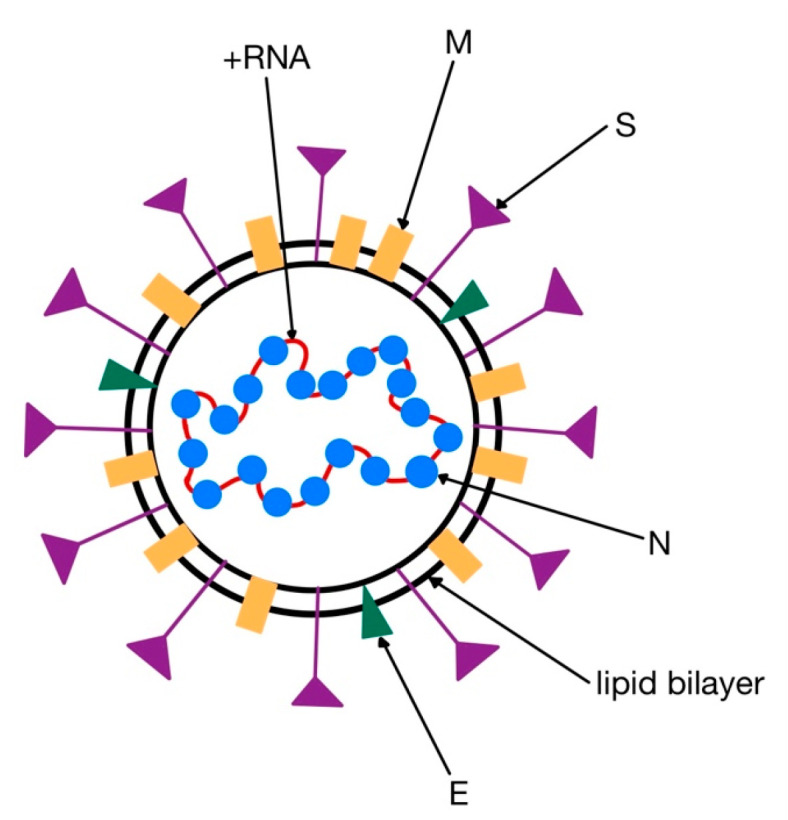
Structure of human coronaviruses. S, spike protein; E, envelope protein; M, membrane protein; N, nucleocapsid protein; +RNA, genome (adapted after [32]).

Coronaviruses are enveloped viruses, with a positive single stranded ribonucleic acid (ss (+) RNA) genome. They contain four major structural proteins: spike (S), membrane (M) and envelope (E)—located in the envelope, and nucleocapsid (N)—located in the ribonucleic acid core (Figure 3) [18,29].

The distinctive “spikes” on the surface (that give the virus a ”crown”-like appearance—hence the name ”corona”, Latin for crown) are trimers of S proteins, which represent class I viral fusion proteins. They bind to cell receptors and mediate the fusion of the virion with the cytoplasmic membrane of the host cell. HCoVs use different receptors to enter the cell: while both SARS-CoV and SARS-CoV-2 bind to ACE2, MERS-CoV uses dipeptidyl peptidase 4 (DPP-4 or CD26) [32,34,35]. The S protein can be partially or completely cleaved by furin-like proteases into two polypeptides, S1 (that contains the receptor binding domain, RBD) and S2 [32,36].

The most abundant protein, however, is the M protein, that gives the envelope its shape. Cryo-electron microscopy revealed this molecule to associate in dimers. The E protein is only found in reduced amounts in the envelope, and is critical for viral infectivity. The N protein is the sole protein that forms the helical nucleocapsid around the genome. This phosphoprotein binds to both the RNA and the M protein, in order to maintain the structure of the viral core [36].

In addition to the four canonical proteins, some strains of HCoVs also have a haemagglutinin-esterase protein (HE), that forms a secondary set of short projections in a similar manner to the S protein. This molecule (similar to the haemagglutinin of orthomyxoviruses) has both the ability to bind to and cleave sialic acid, acting as a cofactor for the S protein by assisting the attachment of the virus to host cells and facilitating its travel through the mucus layer [32,37].

RNA viruses are more susceptible to replication errors (mediated by the RNA polymerase) than DNA viruses [38]. Therefore, various mutations localized on the S protein led to the emergence of new strains, each one with modifications in viral pathogenesis, such as changes in the RBD and novel escape capabilities from natural immunity [39,40,41]. The Omicron variant is characterized by over 30 mutations in the spike protein, resulting in a higher transmission rate and significant immune evasion, both from natural immunity and the currently used vaccines [38,40,42,43].

## 5. Viral Entry and Replication

**Figure 4:**
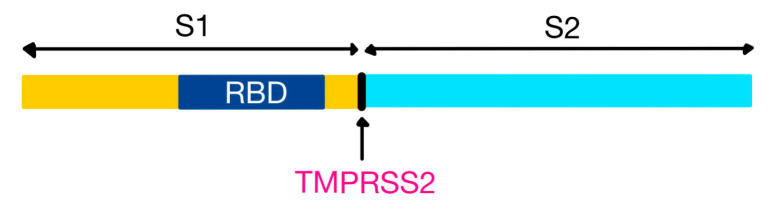
SARS-CoV-2 spike (S) protein domain structure (with two subunits, S1 and S2) and cleavage sites. TMPRSS2, transmembrane serine protease 2 (adapted after [45]).

SARS-CoV-2 uses angiotensin-converting enzyme 2 (ACE2) as a receptor for entry into the host cell. However, the entry is dependent on an accessory furin-like molecule: the transmembrane serine protease 2 (TMPRSS2)—also expressed in pneumocytes, necessary for cleaving the viral S protein at the S1/S2 site (Figure 4) [36,44,45,46,47].

The S protein is functionally similar to fusion proteins of other viruses (orthomyxoviruses, human immunodeficiency virus—HIV and Ebola virus). However, its two functional components need to be separated to complete viral entry [18,36].

**Figure 5:**
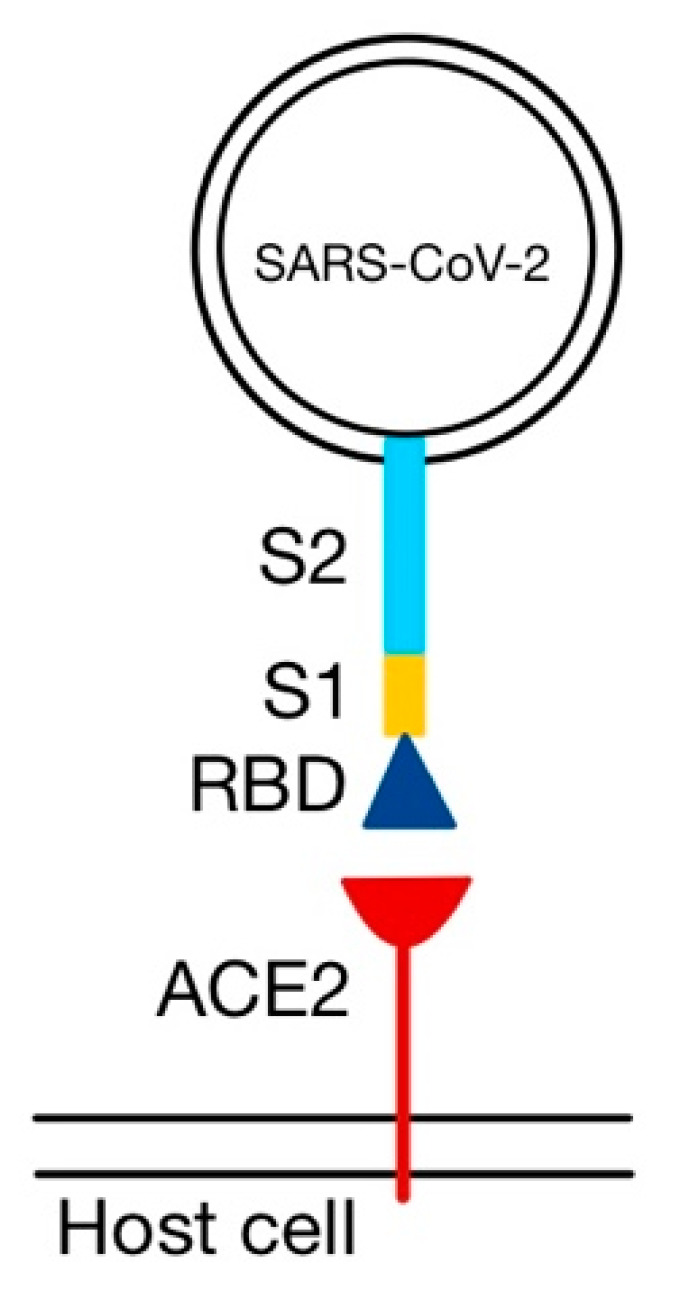
The interaction between the receptor binding domain (RBD) of the S1 subunit and angiotensin converting enzyme 2 (ACE2) (adapted after [45]).

S1 binds to ACE2 via its RBD (Figure 5), and S2 facilitates the juxtaposition of cellular and viral membranes, to allow the mixing of their lipid bilayers and the discharge of the contents of the virion into the cytoplasm [36,48,49]. SARS-CoV-2 binds to ACE2 with higher affinity than SARS-CoV, because the RBD of the S protein is usually hidden from the surface (unlike the S protein of SARS-CoV), enhancing its ability to evade the immune system [18,50,51].

**Figure 6:**
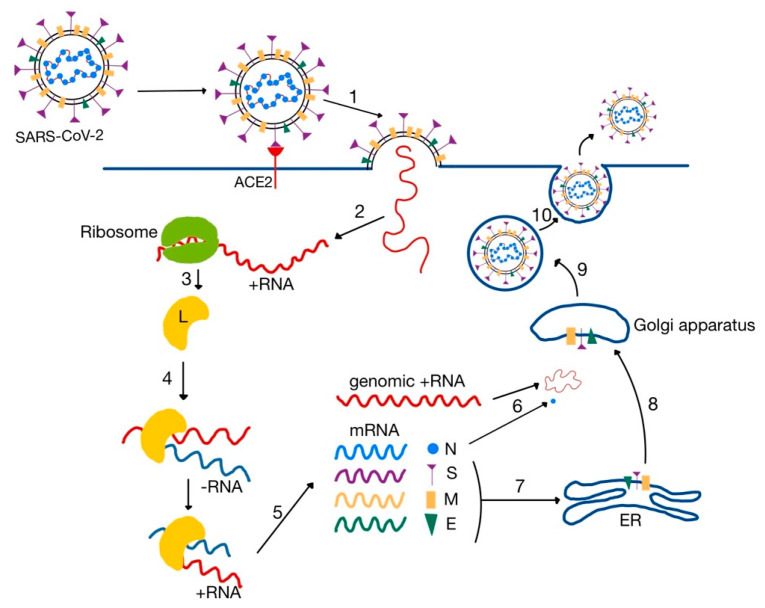
SARS-CoV-2 entry and replication cycle. L, RNA polymerase; ER, endoplasmic reticulum. 1—binding to ACE2 and viral entry via endocytosis; 2—release of viral genome; 3—translation of L protein; 4—RNA replication; 5—transcription of viral genome and proteins; 6—translation of N protein and assembly of nucleocapsid in cytoplasm; 7—translation of S, M and E proteins in the ER; 8—combining of S, M and E proteins with the nucleocapsid in the Golgi body; 9—formation of mature virion; 10, exocytosis (adapted after [32]).

After the fusion of the membranes, the genome is translated to produce an RNA-dependent RNA polymerase (L). The L protein uses the genomic (+) RNA to transcribe a complementary (−) RNA chain [30]. The (−) RNA acts as a template for the synthesis of both new genomic (+) RNA molecules and messenger RNA (mRNA). The mRNA is then translated to synthesize the viral proteins: M, E, S and N. The three envelope proteins (M, E, S) are inserted into the endoplasmic reticulum (ER), where they are packed into vesicles and transported to the Golgi apparatus. Here, they form the envelope around the nucleocapsid core, which is composed of newly synthesized N proteins and genomic (+) RNA molecules. The signal for viral assembly was initially thought to be the M protein, but more recent studies emphasized the need of the additional co-expression of both E and N proteins. Finally, the new virions are secreted from the host cell by exocytosis (Figure 6) [32,36,52].

However, a fraction of S proteins remains in the cytoplasm of the infected cell, where it can mediate the fusion of the host cell with adjacent uninfected cells, leading to the formation of multinucleate syncytia [36].

## 6. Ang II-AT_1_R Interaction

The attachment of the S1 subunit of the viral S protein to ACE2 induces the endocytosis of ACE2 together with the virus. As ACE2 controls the anti-inflammatory arm of RAAS, its diminished surface expression leads to an increase of serum Ang II levels, which acts not only as a vasoconstrictor, but also as a pro-inflammatory cytokine via AT_1_R [18,46].

**Figure 7:**
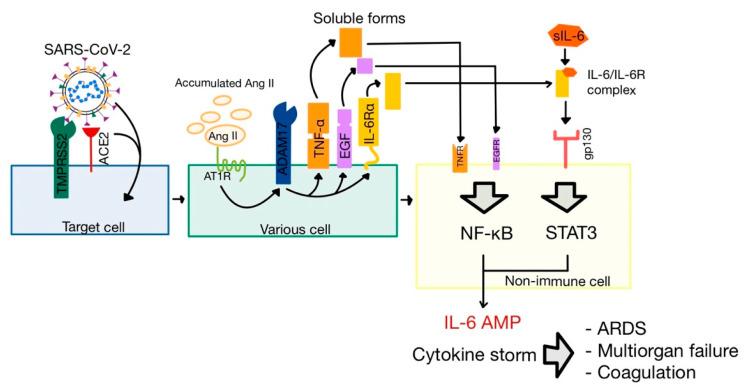
Effects of the interaction between increased Ang II levels and AT_1_R. TNFR, TNF-α receptor; EGFR, EGF receptor; IL-6 AMP, IL-6 amplifier; ARDS, acute respiratory distress syndrome. (adapted after [46]).

The Ang II-AT_1_R signaling axis activates ADAM metalloprotease domain 17 (ADAM17), also named tumor necrosis α converting enzyme (TACE) and expressed on respiratory epithelial cells [32,53,54]. This enzyme has three important actions related to the pathophysiology of the SARS-CoV-2 infection (Figure 7).

1. The cleaving of ACE2 results in the secretion of soluble ACE2 molecules (sACE2). Though the biological function of sACE2 is still unknown, it was successfully used as a treatment in severe cases of COVID-19 [32,36].
2. The cleaving of both tumoral necrosis factor α (TNF-α) and epidermal growth factor (EGF) leads to the stimulation of nuclear factor κ-light-chain enhancer of activated B cells (NF-κB) pathway, one of the major pathways of inflammation [18,46].
3. The cleaving of membrane-bound interleukin-6 receptor α (IL-6Rα) forms soluble IL-6Rα (sIL-6Rα), which interacts with IL-6. The IL-6-sIL-6Rα complex binds to glycoprotein 130 (gp130, also known as IL-6Rβ), activating another one of the major inflammation pathways, JAK/STAT_3_ (Janus kinase/signal transduction and activator of transcription 3) [46,55].

The interaction between Ang II and AT_1_R also increases vascular permeability by increasing the secretion of vascular endothelial growth factor (VEGF) and the expression of endothelial adhesion molecules, including selectins, vascular cell adhesion molecule-1 (VCAM-1), intercellular adhesion molecule-1 (ICAM-1), and their ligands, integrins [18,56].

Another major pathway for Ang II-AT_1_R signaling is the stimulation of NADH oxidases (NOX) to produce reactive oxygen species (ROS) like superoxide (O^2−^) and hydrogen peroxide (H_2_O_2_). ROS amplify the inflammatory actions of the innate immune system [18,57].

## 7. Inflammatory Response and ARDS

**Figure 8:**
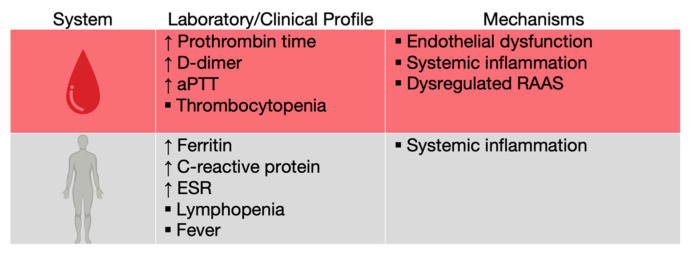
Laboratory and clinical profiles and key mechanisms of systemic inflammation and hyp-ercoagulation in COVID-19. aPTT—activated partial thromboplastin time; ESR—erythrocyte sedimentation rate (adapted after [58]).

COVID-19 patients were observed to have elevations in traditional biochemical markers of acute infection, such as C reactive protein (CRP), ferritin and erythrocyte sedimentation rate (ESR), as well as lymphopenia, probably due to T cell redistribution to the lungs, exhaustion, or depletion through TNF-α-mediated apoptosis (Figure 8) [58,59,60].

However, in severe forms of the disease, patients may be in a hyperinflammatory state, leading to lung damage and even acute respiratory distress syndrome (ARDS) [58].

Upon viral infection, pneumocytes, alveolar macrophages and circulating monocytes are activated by the interaction of pathogen-associated molecular patterns (PAMPs), like the viral RNA, with cellular receptors called pathogen recognition receptors (PRRs)—the most important being Toll-like receptors (TLRs) [32,46]. Single stranded RNA is recognized by TLR7 and TLR8, while TLR3 is activated by double stranded RNA. However, there are other TLRs that take part in viral recognition. TLR2 and TLR4 traditionally recognize bacterial components, such as peptidoglycan, lipoteichoic acid or lipopolysaccharide. On the other hand, recent studies have demonstrated that TLR2 and 4 can also be triggered by damage-associated molecular patterns (DAMPs)—debris from infected dying cells, like histones and oxidated phospholipids [22,61,62,63]. Activated PRRs determine the production of inflammatory cytokines and chemokines, which attract more immune cells (especially monocytes and T lymphocytes), resulting in a widespread inflammation of the lung [46,64].

The apoptosis of infected type 2 pneumocytes induce the release of inflammatory mediators, that activate alveolar macrophages to secrete three essential cytokines: IL-1, IL-6, and TNF-α [32,65]. IL-6 interacts with its receptor (whose concentration was increased following the activation of ADAM17 by Ang II), in order to activate JAK/STAT3, and TNF-α is cleaved by ADAM17 and stimulates the NF-κB pathway. The simultaneous activation of JAK/STAT3 and NF-κB leads to a positive feedback loop of hyper NF-κB activation (named “the IL-6 amplifier”), which results in a colossal production of cytokines, known as the cytokine release syndrome (CRS) or “cytokine storm”. This may be the reason why lung inflammation can, in severe cases, be followed by ARDS, with multi-organ failure and coagulation (Figure 7) [46,66,67].

Apart from the three cytokines, activated macrophages secrete chemokines, including CCL2 (monocyte chemoattractant protein 1—MCP1), CCL5, CXCL8 (IL-8) and CXCL10. These molecules stimulate the recruitment of neutrophils and natural killer (NK) cells to the affected lung parenchyma. High levels of ROS produced by neutrophils overwhelm endogenous antioxidant systems, resulting in oxidative stress and cell injury. NK cells secrete granzymes (proteases) and perforins, hence producing both pro-apoptotic and pro-necrotic effects. Excessive NK cytotoxicity determines uncontrolled lung damage. These effects also contribute to ARDS, alongside CRS [68,69,70].

ARDS is a life-threatening form of respiratory failure, a common complication of COVID-19. It was first defined in 1968, its symptoms including acute hypoxemia, non-cardiac pulmonary oedema and low pulmonary compliance. Studies conducted during the COVID-19 pandemic showed that 33% of hospitalized patients infected with SARS-CoV-2 developed ARDS, while 75% of patients admitted to the intensive care unit had ARDS [25,68,71].

## 8. Coagulation

Although most patients infected with SARS-CoV-2 suffer from lung damage, nearly 70% of the hospitalized ones develop coagulopathies, such as hypercoagulation, disseminated intravascular coagulation and venous thrombosis [72,73]. Early reports from Wuhan, China showed a prolonged activated thromboplastin time (aPTT) and prothrombin time, elevated D-dimer (a fibrin degradation product) levels and thrombocytopenia, suggesting a level of hypercoagulability (Figure 8) [58,74].

**Figure 9:**
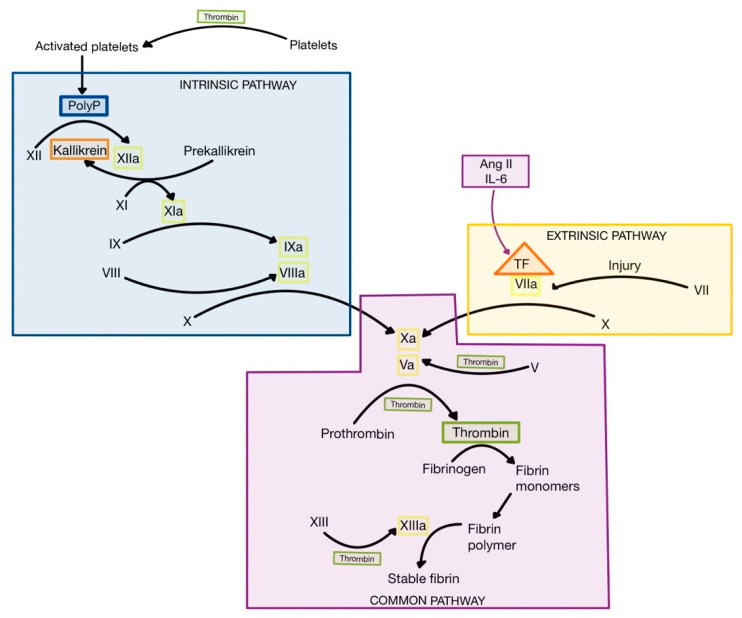
The coagulation cascade. PolyP, polyphosphate; TF, tissue factor (adapted after [1]).

Ang II can induce the synthesis and expression of tissue factor (TF), a proteolytic enzyme made up of phospholipids from tissue membranes and a lipoprotein complex, which is released following injury and inflammation from various cell types, including endothelial cells, pneumocytes, fibroblasts, macrophages and neutrophils [3,75]. Also, CRS leads to high levels of IL-6, which induces expression of TF on monocytes, macrophages and endothelial cells [76,77]. When TF is released, it interacts with factor VII to form activated factor VII (VIIa), initiating the extrinsic pathway of coagulation (Figure 9) [1,3]. The result is the formation of thrombin (factor IIa) from prothrombin (factor II) [1,75].

Thrombin is the central protease of the coagulation cascade, with multiple actions. Firstly, thrombin turns fibrinogen into fibrin monomers, which polymerize spontaneously to form fibrin fibers, that trap blood cells. Secondly, thrombin activates factor XIII (fibrin stabilizing factor—FSF), which cross-links fibrin fibers to form a mesh named stable fibrin. Thirdly, thrombin interacts with the protease-activated receptor 1 (PAR-1), a G protein-coupled receptor expressed on thrombocytes, to promote platelet activation. Platelets release polyphosphate (PolyP) stored in granules, which represents an activator of factor XII (Hageman). The formation of factor XIIa initiates the intrinsic pathway of coagulation, leading to the activation of the coagulation cascade and the formation of even higher levels of thrombin. In addition to its procoagulant role, factor XIIa can also contribute to inflammation by activation of plasma kallikrein (Figure 9), resulting in the formation of bradykinin, a proinflammatory peptide [1,75,78].

**Figure 10:**
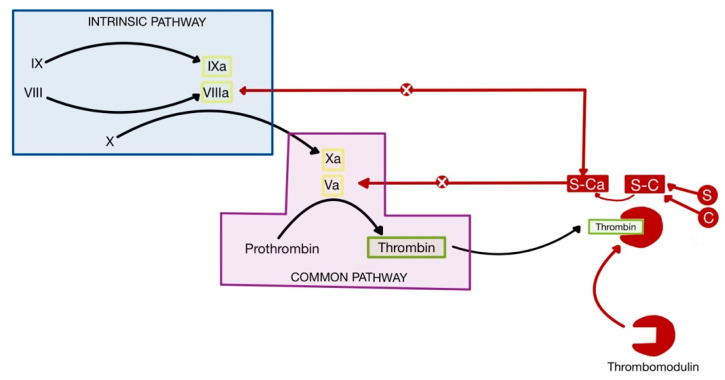
Physiological anticoagulants. S-C denotes the interaction of protein C with its cofactor, protein S (adapted after [1]).

This mechanism contributes to increased thrombosis in COVID-19, in addition to the higher concentration of IL-1 and TNF-α produced by CRS, which leads to the suppressed activity of physiological anticoagulants (Figure 10) [75,77]. Thrombomodulin is a glycosaminoglycan produced by endothelial cells, which forms a complex with thrombin, in order to remove it from circulation and inhibit coagulation. Protein C binds to the thrombomodulin-thrombin complex and is activated, forming active protein C (Ca). Ca, together with its cofactor, protein S, inactivates factors Va and VIIIa, thus inhibiting coagulation. IL-1 and TNF-α decrease the availability of thrombomodulin, hence suppressing the anticoagulant pathway [1,75,79,80].

Also, increased IL-6 levels stimulate the hepatic production of thrombopoietin (TPO), which leads to thrombocytosis. Paradoxically, severe cases of COVID-19 are characterized by thrombocytopenia [75,81]. There are three possible mechanisms:

1. destruction of bone marrow progenitor cells by the cytokine storm, leading to a decrease in platelet production;
2. immune platelet destruction by autoantibodies;
3. thrombosis in the lungs, leading to platelet consumption [82].

## 9. Systemic Manifestations

**Figure 11:**
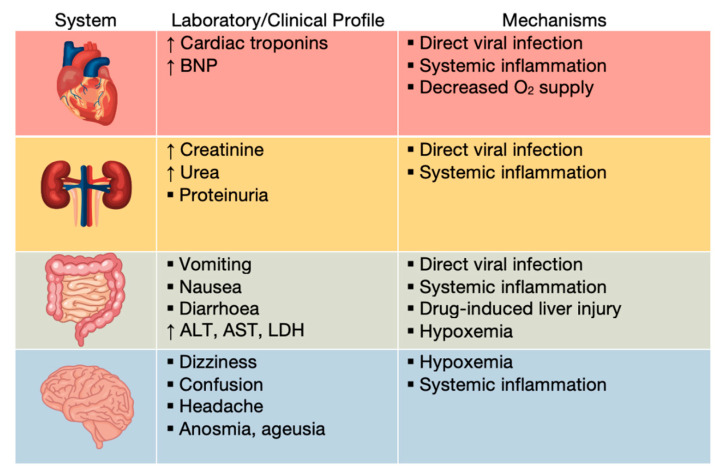
Laboratory and clinical profiles and key mechanisms of systemic manifestations of COVID-19.O_2_—oxygen; BNP– brain natriuretic peptide; ALT—alanine transaminase; AST—aspartate transaminase; LDH—lactate dehydrogenase (adapted after [58]).

Severe cases of COVID-19 can progress to systemic disease, characterized by multisystem organ damage or failure (Figure 11) [58].

### 9.1. Cardiovascular Manifestations

While respiratory failure dominates the early phases of disease, cardiac injury becomes critical during later phases [83].

Patients with severe COVID-19 were demonstrated to have elevated levels of cardiac troponin and brain natriuretic peptide (BNP), classical markers of cardiac injury. Increasing troponin levels have been correlated with other inflammation markers, such as C reactive protein (CRP), ferritin and IL-6, suggesting inflammatory cardiac damage, as opposed to primary injury. CRS directly affects cardiomyocytes, causing myocarditis without direct viral infection, as well as arrythmias, such as supraventricular or ventricular tachyarrhythmia and bradyarrhythmia [58,83,84,85,86]. Invasion of the virus in myocytes is also possible. ACE2 expression is elevated in the cardiac tissues, facilitating myocarditis due to direct infection [87,88].

The pathogenesis of myocarditis and cardiac arrhythmias is not currently known. However, several mechanisms were proposed:

1. direct myocardial injury (by either CRS or direct infection), causing a disruption in electrical conduction;
2. pericardial inflammation, resulting in massive edema;
3. pericyte damage in the cardiac micro-vasculature, leading to ischemia;
4. scars due to post-inflammatory fibrosis;
5. gap junction dysfunction, as a result of pro-inflammatory cytokines (e.g. IL-6) [85,89].

Ischemic cardiac injury can occur in patients with or without coronary artery disease (CAD). In patients that suffer from CAD, the primary cause is either plaque rupture (because of the destabilization of atheromatous plaques due to the activation of inflammatory cells by circulating cytokines), or thrombosis, as suggested by the hypercoagulative state of patients [83,87,89]. In patients without CAD, myocardial injury is a cause of the discrepancy between myocardial oxygen supply and demand, a situation known as type 2 myocardial infarction. Pulmonary affectation and associated hypoxemia reduce oxygen supply to the heart, while systemic infection and fever increase metabolic needs of peripheral tissues, hence elevating the demand for oxygen of the cardiomyocytes [88,89].

The important role of the vascular endothelium and endothelial dysfunction in the pathogenesis of SARS-CoV-2 infection has been highlighted in the literature, and several interrelated mechanisms involving the RAAS have been discussed in the context of the pandemic.

The vascular endothelium consists of a single layer of cells lining the interior of blood vessels. It is responsible for regulating oxidative stress by releasing mediators such as nitric oxide (NO), endothelin, prostacyclin and controlling the local activity of angiotensin II. It acts as a semipermeable membrane, essential for maintaining the regulating of vascular tone, maintaining the balance between fibrinolytic and procoagulant factors, and also for vascular homeostasis [86].

Endothelial dysfunction (ED) is definite by an alteration in the regulatory functions of the endothelium, causing an imbalance between anticoagulant and procoagulant mediators. Pathophysiological mechanisms of endothelial dysfunction include a combination of risk factors for cardiovascular disease: hypertension, hypercholesterolemia, hyperglycemia, tobacco use, and also hyperhomocysteinemia [86,87].

The cardiac injury in COVID-19 may also be exacerbated due to the dysfunction of the nitric oxide (NO) system. NO is extremely important in the control of blood flow and blood pressure. NO is synthesized by three isoforms of NO synthases (NOS): endothelial (eNOS), neuronal (nNOS) and inductible (iNOS). This enzyme cleaves L-arginine into citrulline and NO, which diffuses to nearby smooth muscle cells and activates the soluble guanylyl cyclase, that converts guanosine triphosphate (GTP) to cyclic guanosine monophosphate (cGMP). cGMP activates the cGMP-dependent protein kinase (GPK), and the enzyme phosphorylates the myosin light chain kinase (MLCK) and the sarcoplasmic/endoplasmic reticulum Ca^2+^-ATP-ase (SERCA). Thus, GPK inhibits MLCK, stopping the interaction between actin and myosin, and activates SERCA, decreasing the intracellular Ca^2+^. The result is vascular smooth muscle relaxation, leading to vasodilation [1]. In normal conditions, Ang 1–9 stimulates the release of bradykinin, thus increasing NO availability. However, in COVID-19, ACE2, which forms Ang 1–9, is downregulated, leading to decreased levels of NO. This may contribute to the suppression of the key roles of NO, such as vasodilation, which decreases the oxygen supply of cardiomyocytes and promotes ischemic cardiac injury [59,87].

### 9.2. Renal Manifestations

ACE2 is expressed in renal cells, including podocytes, mesangial cells, epithelium of Bowman’s capsule and tubular epithelium. Hence, direct infection of these cells is possible, frequently resulting in proteinuria and a minor serum creatinine elevation [58,88,90]. On the other hand, CRS, thrombosis and hypoxemia can lead to acute kidney injury (AKI), estimated to affect 20–40% of critically ill patients in intensive care [58,83,91].

### 9.3. Gastrointestinal and Hepatic Manifestations

Digestive symptoms of COVID-19 include loss of appetite, diarrhea, nausea and vomiting. There is evidence of direct SARS-CoV-2 infection of the enterocytes, due to the abundance of ACE2 receptors in the lower gastrointestinal tract [58,83,92,93].

Apart from milder symptoms such as ageusia, despite a high ACE2 expression in the lingual mucosa and gustatory papillae, the oral cavity does not suffer extensive damage in COVID-19. However, the presence of SARS-CoV-2 in the saliva contributed to the discovery of an important diagnostic tool. ACE2 expression in the minor salivary glands was recently found to be even higher than in the pulmonary system, concluding that saliva testing may be a better noninvasive diagnostic method [25,26].

Patients may also present liver injury, likely because of the direct cytopathic effect of the virus, ARDS, hypoxemia or drug-induced liver injury. The hepatocellular damage is characterized by steatosis, inflammation, and even necrosis, leading to hepatic enzyme abnormalities, such as elevated lactate dehydrogenase (LDH), aspartate transaminase (AST) and alanine transaminase (ALT) [37,58,83,88,94,95].

### 9.4. Central Nervous System Involvement

Even though ACE2 receptors are present in the cerebral cortex and brain stem, there is no definite evidence of direct injury of the central nervous system by SARS-CoV-2 [83,88]. Altered oxygen levels (hypoxia caused by the acute pulmonary infection) may contribute to the most common symptoms, such as dizziness, headache, confusion and delirium [83,96,97].

The hyperinflammatory status and cytokine storm can cause brain inflammation and edema, as cytokines can pass directly through the blood-brain barrier. Thrombotic events and endothelial injury may be responsible for strokes [83,88,98,99].

Anosmia and ageusia are frequently reported, but their causes are still unknown. A potential explanation is the presence of ACE2 receptors in the olfactory and gustatory pathways, as MRI studies showed abnormalities in the olfactory bulbs and the posterior part of gyrus rectus [88,96,100,101].

### 9.5. Oral Manifestations

Patients diagnosed with SARS-CoV-2 can present various clinical symptoms, specific or non-specific, sometimes asymptomatic. The SARS-CoV-2 virus was detected in the patient’s saliva, which could cause lesions at this level, as in many viral diseases [102,103,104,105]. In addition to affecting the respiratory, cardiovascular, renal, neurological, and gastrointestinal systems, data from the literature also exemplified the presence of oral manifestations such as salivary gland infections, erythema, aphthous, ulcers, gingivitis, taste disorders, and also xerostomia [106]. These were correlated with the presence of risk factors such as smoking, poor oral hygiene, pre-existing dental conditions such as periodontal disease, associated comorbidities, and also with the medication administered. The data from the literature demonstrated that acute parotitis and secretory dysfunction of the salivary glands can be initial symptoms of COVID-19 [107].

The etiology involved in oral manifestation associated with the infection with COVID-19 remains controversial. At the present, there is not enough data to support the hypothesis that these lesions are directly caused to SARS-CoV2 (through the inflammatory effects and tissue destruction produced at the oral level) or this could be associated with SARS-CoV-2 infection together with favorable factors, and adverse reactions to drugs, damage to the immune system [108].

Moreover, it is difficult to analyze the etiology and the association between oral manifestations and the COVID-19 pandemic [109]. It remains an important subject to analyze and discuss whether oral manifestation is directly caused by SARS-CoV-2 or secondary manifestations.

## 10. Conclusions

SARS-CoV-2 binds to ACE2 receptors present throughout the organism, causing an inflammatory response. However, this interaction downregulates ACE2, inhibiting the anti-inflammatory arm of RAAS and exacerbating the pro-inflammatory effects of Ang II, leading to further production of inflammatory cytokines and promoting a cytokine storm.

As a result, besides pulmonary lesions, hypercoagulation and ARDS, the repercussions of this emerging disease regarding other organs, including the heart, oral cavity, gastrointestinal tract, liver, kidneys and brain, must be taken into account in order to efficiently manage the global pandemic. Often, ARDS and multiorgan failure lead to death, hence explaining the high mortality of COVID-19.

Oral manifestation and COVID-19 remain an important issues to analyze and discuss because the prevalence of these manifestations is still unknown.

The number of cases patients reported is few in comparison with the number of SARS-CoV-2 infection patients diagnosticated in the world.

Moreover, it is necessary to have more detailed studies and complementary tests in these patients such as hematological tests/ biopsies to identify the possible etiopathogenesis or factors that influence these oral lesions. Also, it is necessary to include dentists in the management of patients with SARS-CoV2 infection for a careful oral examination to diagnose, treat, and see the evolution of the lesions described.

In conclusion, a deeper understanding of the interrelationship between the SARS-CoV-2 virus-type 2 angiotensin converting enzyme—severe multi-organic complications opens the way to research and the discovery of new therapies for the treatment of this infection which, through the various forms of ailments that wears them, led to the outbreak of a pandemic and the emergence of a severe health crisis worldwide.

## Data Availability

Not applicable.
